# Progress in Surgery

**Published:** 1900-09-29

**Authors:** 


### Progress in Surgery.

### SURGERY OF THE INTESTINES.

**Ulceration and Perforation.** — Taylor[1] reports five cases of operation for typhoid perforation, only one of which recovered. The author states that less than two hundred cases in all have been operated on, with still a very heavy mortality, but that nothing short of a moribund condition should warrant us in abandoning the case as hopeless. The key to success is in early operation. Keen[2] summarises his opinions on the surgical treatment of perforation of the bowel as follows: (1) The surgeon should be called in consultation the moment any abdominal symptoms indicative of possible perforation are observed. (2) If it be possible to determine the existence of the perforative stage, exploratory operation should be done under cocain anæsthesia before perforation, shock, and sepsis have occurred. (3) After perforation has occurred, operation should be done at the earliest moment, provided (4) that we wait till the primary shock, if any be present, has subsided. (5) If in a case of suspected but doubtful perforation, a small exploratory opening should be made, under cocain, to determine the existence of a perforation; and if hospital facilities for a blood-count and for immediate bacteriological observation exist, then surgical aid should be invoked. (6) The operation should be done quickly, but thoroughly, and in accordance with the modern technique. (7) The profession at large must be aroused to the possibility of a cure in nearly, if not quite, one-third of the cases of perforation, provided speedy surgical aid is invoked. Perkins and Wallace[3] relate a successful case of operation for duodenal ulcer on a man aged 52 years. Ten hours after the onset of acute pain the abdomen was opened through the right rectus, the age and sex of the patient, the history, and the absence of liver dulness making it probable that a duodenal ulcer had perforated. A perforation was discovered at the junction of the first and second parts of the duodenum; the edges were clean cut and showed no signs of induration. The perforation was closed with two rows of sutures, and the abdomen cleaned after evisceration and flushing with sterilised water. The abdominal opening was closed with silkworm gut sutures, but a gauze wick was left in passing down to the seat of the ulcer without further drainage. The plug was removed on the second day.

**Intestinal Obstruction.**—Parker Lyons[4] says there are two main classes of acute obstruction: (1) Those in which there is not only an occlusion of the intestinal canal, but a condition of strangulation; (2) those in which the fæcal flow only is arrested. The great majority of cases belong to the first class. The author's conclusions are: (1) Acute intestinal obstruction is a fatal condition, if not promptly and properly relieved; (2) it should rarely be fatal if promptly recognised and treated; (3) acute obstruction is a mechanical condition, and can be relieved only mechanically; (4) the symptoms are absolutely characteristic and distinct, and the diagnosis can always be made, unless the clinical picture has been obscured by the administration of opium; and (5) an early laparotomy, properly performed, is the only form of treatment which should be relied upon in this class of case. Cases of intestinal obstruction due to bands are recorded by Platt,[5] and one due to a persistent Meckel's diverticulum is reported by Fawssett and Jowers.[6] Each case made a successful recovery.

**Artificial Anus.**—Weir's conclusions[7] on this subject are: (1) That an artificial anus of a temporary character can be best established by Maydl's operation or by Bodine's modification; (2) that over-slipping of fæces may be prevented by proper spur formation, by narrowing the rectal opening, or by occluding the rectal end of the bowel, which may be fastened in the wound, or dropped into the abdominal cavity; (3) that continence of the abnormal outlet is aided by muscle separation (Maydl), or by muscle bridging (Von Hacker and Hartmann), or by the use of inflatable or moulded plugs or other apparatus; (4) it is only, however, to be satisfactorily effected (though larger experience in this is desirable) by an extra-abdominal iliac outlet (Witzel's iliac colostomy), to be made by opening the bowel outside and behind the iliac spine. In this procedure the bowel is compressed between the edge of the bony pelvis and the skin.

**Intestinal Anastomosis.**—A new invagination method of anastomosis is described by Morisani,[8] who has employed it successfully in a number of cases. A strip of mucous lining is removed from the inner surface of the distal end of the intestine, so that it permits the proximal to be invaginated into it. When this has been accomplished it is held in place by fixation sutures, while a continuous suture unites the cut end of the distal portion of the gut, with its raw muscular layer, to the serous surface of the proximal or invaginated layer. The process of invagination protects the stitches and the line of union in a degree from contamination by the intestinal contents, while strengthening it against pressure from within. The denuding of the muscular coat where it is brought in contact with the serous surface of the proximal portion in the line of union hastens the separative process and frees the wound from the contamination of the intestinal contents. The histological examinations made on the experimental cases showed that firm adhesion is present in 24 hours, and that there is no contraction of the lumen of the gut. A case of excision of the cæcum for ileo-cæcal cancer, in which anastomosis was effected by a Murphy button, is recorded by Morrison,[9] and one of enterectomy for fæcal fistula by Wallis.[10]

**Murphy Button.**—In the eight years during which the button has been used the following conclusions, says Murphy,[11] have been reached: (1) It approximates without suture; (2) the time of operation is much shortened; (3) the union is ideal; (4) there is no contraction of the scar; (5) the physiological function of the gut is not interrupted at any time. There are two great classes of objections to the use of the button—(1) the opening might be occluded by food or other particles prior to the sloughing of its attachment; (2) there might be prolonged retention of the button in the gut or abdominal cavity. Both these matters were of small moment, there having been but three fatal cases in the 1,620 considered traceable to such causes. It is of little consequence whether the button is passed or not. The portions of the canal in which it has been found retained are as follows: In the stomach, 22 times; colon, 5 times; ileum, 2 times; jejunum, 1 time; cæcum, 2 times; rectum, 4 times. The value of the button is shown by the fact that enterostomy, which formerly yielded a mortality of from 50 to 100 per cent., now shows a mortality of but 19·7 per cent. McArdle,[12] who is an enthusiastic advocate of the use of the button, fully endorses these favourable views.

**Experiments on Intestinal Suture** have been made by Edmunds and Stabb.[13] The methods contrasted were by use of (1) Halsted's inflated rubber cylinders; (2) Murphy's button; (3) Laplace's intestinal forceps. Seven experiments by each method were made on dogs, with the result that all the seven dogs in which the intestine after complete division was reunited circularly by the aid of Halsted's cylinders, recovered; of the seven experiments with Murphy's button, only five were successful; and of the seven with Laplace's forceps, only four were successful. The failures were all due to non-union. The verdict appears in favour of Halsted's inflated rubber cylinders. The effect of the inflated cylinder is to push back the mucous membrane, which otherwise always everts itself so much as to interfere materially with the suturing of the outer coats and the adaptation of the peritoneal surfaces. The cylinder also prevents the escape of the intestinal contents, thus dispensing with clamps of any kind. Murphy's button does not appear to be trustworthy, unless external sutures are applied; but if this is done, the time of the operation is lengthened, and the instrument is in some degree reduced to an apparatus for facilitating the application of sutures. Laplace's forceps afforded no aid in suturing, and, as above stated, three out of the seven junctions made with its use failed.

**Intussusception.**—Kammerer[14] thinks that of the bloodless methods of treatment the injection of fluids by the rectum is the only one which deserves serious consideration; but the method is only applicable to the colic and ileo-cæcal varieties. He lays down the following rules for its use: (1) In acute cases an attempt at reduction should only be made very early in the case, and once only; (2) this attempt should always be made under complete anæsthesia, with relaxed abdominal walls; (3) hot water should be employed in preference to iced water; (4) in very acute cases the method should not be employed; (5) after one failure laparotomy is indicated. The author is of opinion that the mortality from intussusception could be vastly reduced if all cases were operated upon immediately after the diagnosis has been made. He recommends resection either of the entire intussusception or of the intussuscepted portion alone. Gibson[15] states that of 34 cases in which an artificial anus was established the mortality was 83 per cent.; and in 32 cases of resection the mortality was 81 per cent. In only one case of resection of gangrenous intestine did the patient recover. Successful cases of laparotomy for intussusception are reported by Farmer[16] and Lediard.[17]

[1] Clinical Jour., May 30, 1900, p. 90. [2] The Post Graduate, Feb., 1900, p. 272. [3] Lancet, Feb. 17, 1900, p. 458; and Brit. Med. Jour., Feb. 17, 1900, p. 383. [4] Med. Rec., Mar. 3, 1900, p. 389. [5] Med. Chron., May, 1900, p. 102. [6] Lancet, June 2, 1900, p. 1585. [7] Med. Rec., April 21, 1900, p. 667. [8] Amer. Jour. Med. Sci., Feb., 1900, p. 219. [9] Birmingham Med. Rev., April, 1900, p. 193. [10] Clinical Jour., June 13, 1900, p. 125. [11] Med. Rec., June 9, 1900, p. 1003. [12] Dublin Med. Jour., Mar., 1900, p. 161. [13] Lancet, April 14, 1900, p. 1067. [14] Birmingham Med. Rev., April, 1900, p. 241. [15] Ibid., p. 243. [16] Brit. Med. Jour., May 26, 1900, p. 1284. [17] Lancet, Feb. 17, 1900, p. 454.

